# Antioxidant and Compositional HPLC Analysis of Three Common Bamboo Leaves

**DOI:** 10.3390/molecules25020409

**Published:** 2020-01-18

**Authors:** Ning-Hui Ma, Jing Guo, Si-Han Xu Chen, Xiu-Rong Yuan, Tong Zhang, Yue Ding

**Affiliations:** 1 School of Pharmacy, Shanghai University of Traditional Chinese Medicine, Shanghai 201203, China; mnh2029@163.com (N.-H.M.); guojing0915@yeah.net (J.G.); Xuchensihan216@163.com (S.-H.X.C.); 2 Technology Transfer Center, Shanghai University of Traditional Chinese Medicine, Shanghai 201203, China; yuany@189.cn; 3 Experiment Center for Teaching and Learning, Shanghai University of Traditional Chinese Medicine, Shanghai 201203, China; zhangtdmj@hotmail.com

**Keywords:** *Phyllostachys nigra*, *Lophatherum gracile*, *Pleioblastus amarus*, content, antioxidant activity

## Abstract

Bamboo leaves of *Phyllostachys nigra* (PN), *Lophatherum gracile* (LG), and *Pleioblastus amarus* (PA) are three common herbs in China. In this work, a new high performance liquid chromatography (HPLC) method for the simultaneous determination of seven compounds in bamboo leaves has been developed; and PN, LG, and PA leaves were analyzed. PN showed four times as much chlorogenic acid (CA) than the other two, and contained the most isoorientin (iso-ORI) and isovitexin (iso-VIT) as well. The PA presented the most orientin (ORI) and LG covered a majority of cynaroside (CYN). We measured the antioxidant activity by scavenging the stable 2,2-diphenyl-1-pyridinohydrazinyl (DPPH) free radicals, and found that Luteolin (inhibitory concentration (IC)_50_ = 0.42 µM, LUT) and CYN (IC_50_ = 0.43 µM) showed 2–3 times higher antioxidant activity than iso-ORI (IC_50_ = 0.81 µM), ORI (IC_50_ = 0.84 µM), and other related antioxidant standards such as trolox (IC_50_ = 0.97 µM) and ascorbic acid (IC_50_ = 0.93 µM, VC). Among extracts, PN and PA showed considerable antioxidant activity, which was related well with the contents of CA, iso-ORI, and iso-VIT (*p* < 0.05). This study firstly provides evidence for functional antioxidant compounds of bamboo leaves based on statistical analysis of the HPLC analysis and DPPH assay, and it lays a foundation for its further development or utilization.

## 1. Introduction

China, the world’s leading bamboo producing country, is rich in bamboo resources and has more than 500 genus of more than 500 species of bamboo [1]. The “Chinese Traditional Medicine Resources Guide” lists more than 30 species of medicinal bamboo [2,3] including *Lophatherum gracile* (LG), *Pleioblastus amarus* (PA), and *Phyllostachys nigra* (PN), among others. The medical stems and leaves of *Lophatherum gracile* were usually called “Dan Zhu Ye”, the leaves of *Pleioblastus amarus* were commonly named “Ku Zhu Ye”, and “Zhu Ye” was often used to define the leaves of *Pleioblastus amarus* and other bamboo plants. All three types of bamboo leaves have an extremely long history of medicinal and edible use in China [3]. The LG are the only medicinal bamboo contained in the Pharmacopoeia of the People’s Republic of China (hereinafter referred to as the “Pharmacopoeia”). It was first recorded in “Materia Medica of Southern Yunnan” in the Ming Dynasty. “Compendium of Materia Medica” for the first time, described in detail the morphological characteristics of LG, “Pharmacopoeia” was included in 1963 [4,5]. It has the functions of clearing heat and purging fire, removing annoyance and quenching thirst, as well as anti-inflammatory, antibacterial, antiviral, diuretic, and other functions. It is used to treat fever and thirst, urination, short and astringent pain, and sore tongue [4,6]. PA first appeared in Han’s “Mingyi Bielu”, which has the functions of clearing heat and clearing eyes, removing annoyance and thirst, antibacterial, and lowering blood sugar, among others, and it can treat inflammation, diabetes, cardiovascular diseases, and so on [7,8]. PN firstly appeared in Han’s “Mingyi Bielu” and were recorded as “Dan Zhu Ye”. Ming “Materia medica in South Yunnan” began with “Dan Zhu Ye” as the stems and leaves of LG, which have very good antioxidant and antibacterial effects, as well as great effects on oral pathogens and acute and chronic liver diseases [9,10,11]. However, the above three types of bamboo leaves’ characteristics are so similar that are easy to be confused in use. For example, the bamboo leaves used in the classic famous recipe “Bamboo Gypsum Soup” were not clearly marked, and there currently are no studies on the differences between the three types of bamboo leaves [12]. LG also do not have clearly identification methods of their chemical components that are carried in Pharmacopoeia, so people with botanical experience are needed to distinguish them.

Flavonoids and phenolic acids are natural antioxidants found in plants, and have been reported on bamboo leaves [13,14]. Bamboo leaves contain a lot of flavonoids [15,16,17], such as chlorogenic acid (CA) [18], isoorientin (iso-ORI) [19] and orientin (ORI) [20], isovitexin (iso-VIT), Cynaroside (CYN) [21], Luteolin (LUT) [22,23], and Apigenin (API) [24]. These ingredients have effects on oxidation, diabetes, cardiovascular disease, inflammation, and so on. For example, it can be used for facial mask and skin cream [25], and can also be used for processing pickled foods and so on [26]. Antioxidants isolated from the bamboo leaves have also been approved as a food antioxidant by the Food Additive Standardization Committee of the People’s Republic of China and safely used in the development of food, health care products, medicine, and cosmetics in North America, Asia, and other regions. However, bamboo leaves are too similar to availably distinguish them without an integrated quality control method, and there has been no detailed statistical analysis carried out to date for the functional compounds in bamboo leaves.

In the past year, we have made efforts to explore the correlation between the content of compounds and the antioxidant activity of extracts from the medicinal bamboo leaves of China. Firstly, a new high performance liquid chromatography (HPLC) method for the simultaneous determination of seven compounds was developed. Further, we determined that 26 methanol extracts from the leaves of the bamboo PN, LG, and PA, which grow mainly in southern areas of China, showed obvious differences in composition and content between species. We report here the antioxidant potentials of six flavonoids, one phenolic acid and methanol extracts from bamboo leaves of LG, PN, and PA. Further, the correlation between the content of compounds and antioxidant activity of extracts was investigated by the multivariate statistical analysis method, an important statistical analysis method that has been widely used in medical research [27,28,29]. This study firstly provides evidence for functional antioxidant compounds of bamboo leaves based on statistical analysis of HPLC analysis and 2,2-diphenyl-1-pyridinohydrazinyl (DPPH) assay, and it lays a foundation for its further development or utilization.

## 2. Results

### 2.1. Validation of the HPLC Method

Referring to the chromatograms of HPLC-UV detection, PA (No. 8) was selected to validate the method, including linearity, limit of detection (LOD), limit of quantitation (LOQ), precision, repeatability, stability, and recovery test. To construct calibration curves, linearity was tested at seven different concentrations of standards. Regression equations and correlation coefficients revealed a good linear response for the developed method (*r* > 0.9996), as shown in Table 1. LOD and LOQ were from 0.11 to 1.04 µg·mL^−1^, and 0.06 to 0.35 µg·mL^−1^, respectively. The repeatability, as relative standard deviation (RSD), was assessed by five parallel samples and was in the range of 0.14–3.04%. Table 2 presented the results of precision, stability, and recovery test. Intraday variability measurements of standard solutions were used to determine the precision and injected in six copies (RSD < 3%). The stability was evaluated by measuring and comparing the peak area of one sample at 0, 1, 2, 4, 8, 12, and 24 h. The standard solutions, at three different contents (50%, 100%, and 150%), were added into known extracts in recovery test. Further, the mean recovery was calculated using the following formula:

Recovery (%) = 100 × (amount found − original amount)/amount spiked.(1)

The recovery test results were in the range from 92.90% to 122.32%.

### 2.2. HPLC Analysis of Sample

**Figure 1:**
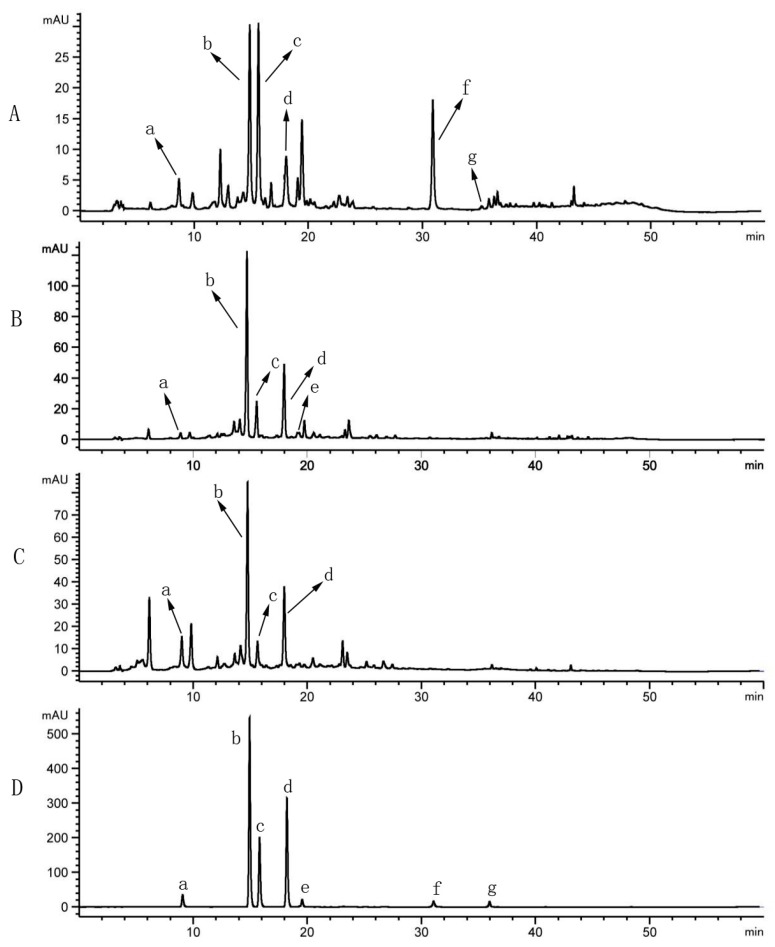
HPLC chromatograms of sample solution and standards. *Lophatherum gracile* (LG, No. 2) leaf-extracts (**A**), *Pleioblastus amarus* (PA, No. 8) leaf-extracts (**B**), *Phyllostachys nigra* (PN, No. 21) leaf-extracts (**C**), and seven mixed reference standards (a, b, c, d, e, f, g) (**D**).

**Figure 2:**
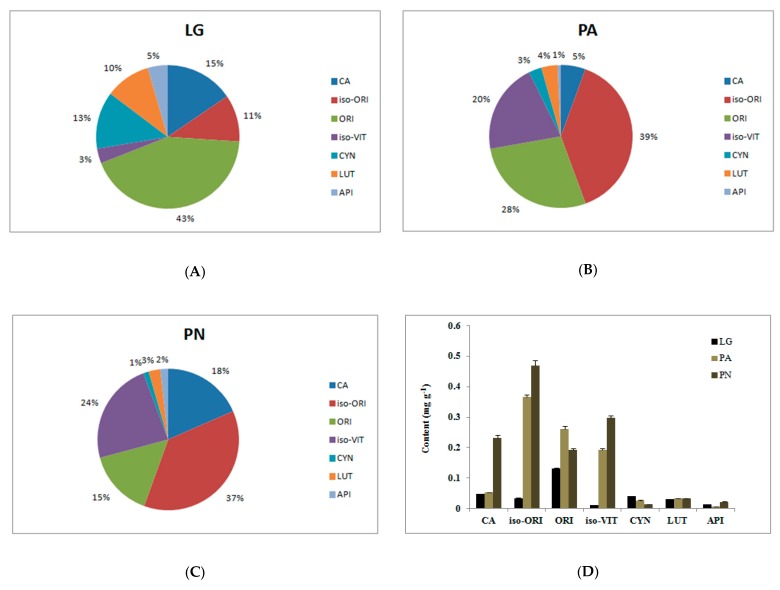
The average relative abundance of compounds in leaves of *Lophatherum gracile* (LG) (**A**), *Pleioblastus amarus* (PA) (**B**), and *Phyllostachys nigra* (PN) (**C**), as well as the comparison of the content of the three specimens (**D**). CA, chlorogenic acid; iso-ORI, isoorientin; VIT, vitexin; CYN, Cynaroside; LUT, Luteolin; API, Apigenin.

Quantitative analysis of seven components was performed on each specimen of bamboo leaves. The method specificity was assessed by comparing the consistency of the retention time between a sample and corresponding reference standard. Figure 1 showed a typical separation of *Lophatherum gracile* (LG), *Pleioblastus amarus* (PA), and *Phyllostachys nigra* (PN) leaf-extracts (A)–(C), and a standard mixture (D) under the optimized chromatographic conditions. All markers were well resolved from background peaks within the analysis time and the seven peaks in the chromatogram of LG, PA, and PN could be identified by the corresponding standards. As listed in Table 3 (results expressed as mean ± SD), PN indicated a four times higher content of CA than the other two, and contained the most iso-ORI and iso-VIT as well. PA presented the highest content of ORI and LG covered a majority of CYN. Figure 2 presented the average relative abundance of compounds in leaves of LG, PN, and PA.

### 2.3. DPPH Assay and Statistical Analysis

**Figure 3:**
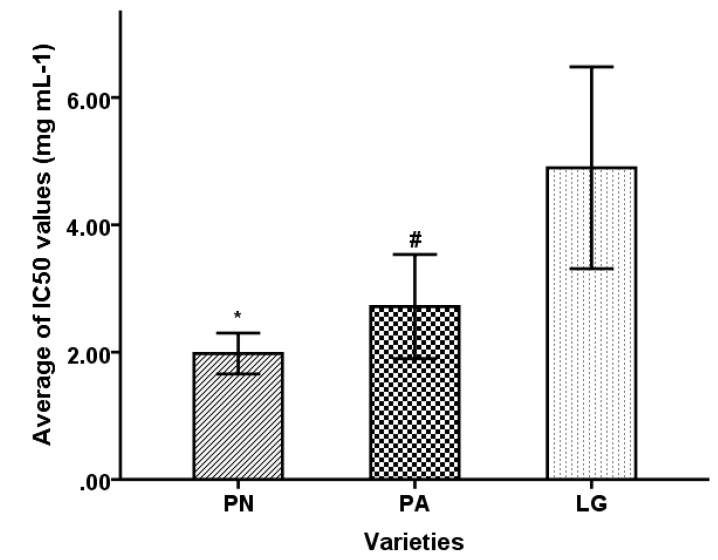
One-way analysis of variance (ANOVA) result of 2,2-diphenyl-1-pyridinohydrazinyl (DPPH) scavenging activity of bamboo leaves from PN, PA, and LG. * means the activity of LG was significantly different from that of PN (*p* < 0.05). ^#^ means the activity of LG was significantly different from that of PA (*p* < 0.05).

Before the DPPH assay, dry ointment yield of extracts was obtained. The antioxidant inhibitory concentration (IC)_50_ values of 26 extracts, 7 compounds, and 2 positive antioxidants were evaluated by scavenging DPPH free radical. These results were listed in Table 4 and Table 5. LUT (IC_50_ = 0.42 µM) and CYN (IC_50_ = 0.43 µM) showed 2–3 times higher antioxidant activity than iso-ORI (IC_50_ = 0.81 µM), ORI (IC_50_ = 0.84 µM), and other related antioxidant standards such as trolox (IC_50_ = 0.97 µM) and ascorbic acid (IC_50_ = 0.93 µM). Among extracts, LG yielded the weakest antioxidant activity, DPPH free radical scavenging IC_50_ values of LG varied from 2.10 to 10.17 mg·mL^−1^, PA and PN showed a considerable and stable ability to scavenge the DPPH free radical (1.25 to 5.07 mg·mL^−1^ and 1.59 to 14.64 mg·mL^−1^, respectively) as shown in Figure 3, and the activity of LG was significantly different from that of PA and PN by one-way analysis of variance (ANOVA) (*p* < 0.05).

As a result of the correlation analysis (Table 5), contents of CA (*r* = −0.508, *p* < 0.01), iso-ORI (*r* = −0.643, *p* < 0.01), and LUT (*r* = 0.431, *p* < 0.05) were significantly correlated with the IC_50_ of PA extracts, the activity of PN extracts was found in relation to the content of ORI (*r* = −0.501, *p* < 0.01), and LG was associated with the content of API (*r* = −0.491, *p* < 0.05). Because of a significant correlation between the content of each component (*p* < 0.05), the stepwise regression method was used in our analysis. We found that the content of iso-ORI and total content of seven compounds jointly explained 53.4% of the variation in the activity of PA extracts, which means iso-ORI is the main active substance in PA. The main antioxidant compounds in PN is ORI (*R*^2^ = 21.9%), and the activity of LG extracts was influenced greatly by the contents of ORI, CYN, and API (*R*^2^ = 95.9%).

**Figure 4:**
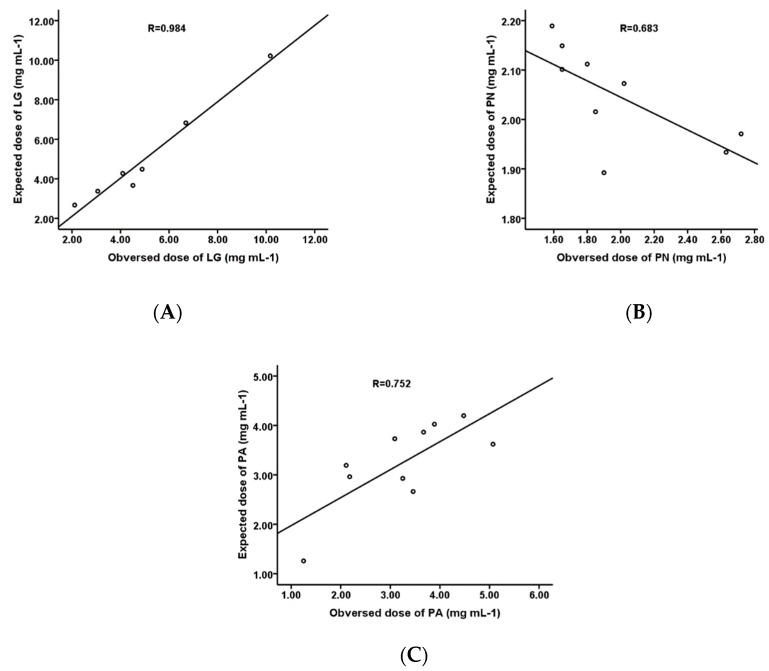
The correlation between observed and expected antioxidant IC_50_ values on LG (**A**), PN (**B**), and PA (**C**). Antioxidant activities were expressed as a dose required for half the DPPH free radical.

Besides, as shown in Figure 4, as a result of correlations between observed and expected antioxidant activities based on established multiple regression equation (Table 6), significant multiple correlation coefficients for DPPH free radical scavenging activities on LG (*R* = 0.984), PA (*R* = 0.752), and PN (*R* = 0.683) were found in relation to the contents of their main active compounds.

## 3. Discussion

Bamboo leaf has been used as an antioxidant material to prevent food deterioration since ancient times. Studies indicated that flavonoids, polysaccharides, and phenolic acids might be the major active constituents [30,31]. Crude extracts and pure compounds from the leaves have been shown to possess multiple biological activities such as anti-inflammatory, antivirus, cardiovascular protection, cancer prevention, and particularly antioxidant activity [32]. However, as far as chemical constituents are concerned, pharmacological reports most often attribute some activities to various extracts rather than specific compounds. This makes it difficult to build a connection between the chemical constituents and pharmacological activities, resulting in the difficulty of identifying the major active compounds. Besides, bamboo leaves are various in varieties and confused in classification, and thus many scientific questions remain, which need to be answered through further laboratory research and clinical trials.

First, we developed a new HPLC method for the quantification of seven main active components in bamboo leaves simultaneously. Several chromatographic conditions using different elution systems were tested according to optimum resolution, peak shape, and efficiency of samples. Conditions were ensured with a Diamonsil Plus C18 column (4.6 × 250 mm, 5 µm) and a water/methanol mixture containing 0.1% (*v/v*) formic acid elution system for the detection of seven compounds. Formic acid (0.1%) was selected as the mobile phase additive to provide improved pH control, simplified preparation, and was found to achieve good peak intensity and resolution. Our preliminary experiments have measured individual maximum UV absorbance of CA, iso-ORI, ORI, iso-VIT, CYN, LUT, and API at 324, 350, 346, 336, 350, 350, 332, and 346 nm, respectively. To achieve optimal UV absorbance for all references, 350 nm was selected and demonstrated a high degree of sensitivity and precision. The method was applied successfully for the analysis and identification of the components of the three bamboo leaves. Then, the antioxidant activity of six flavonoids, one phenolic acid, and methanol extracts from bamboo leaves of LG, PN, and PA were detected by DPPH.

According to Chinese Pharmacopoeia published in 2015, there is no standard assay for determining the contents of LG. In fact, HPLC fingerprints could be used to improve quality control. Assays of biological activities could be applied as well. In addition, studies on this medicinal plant were presently limited to the antioxidants and preservatives, even though ancient books report that the flesh can clear heat and remove vexation. More efforts should be made to fill the blank.

## 4. Materials and Methods

### 4.1. Reagents and Chemicals

**Figure 5:**
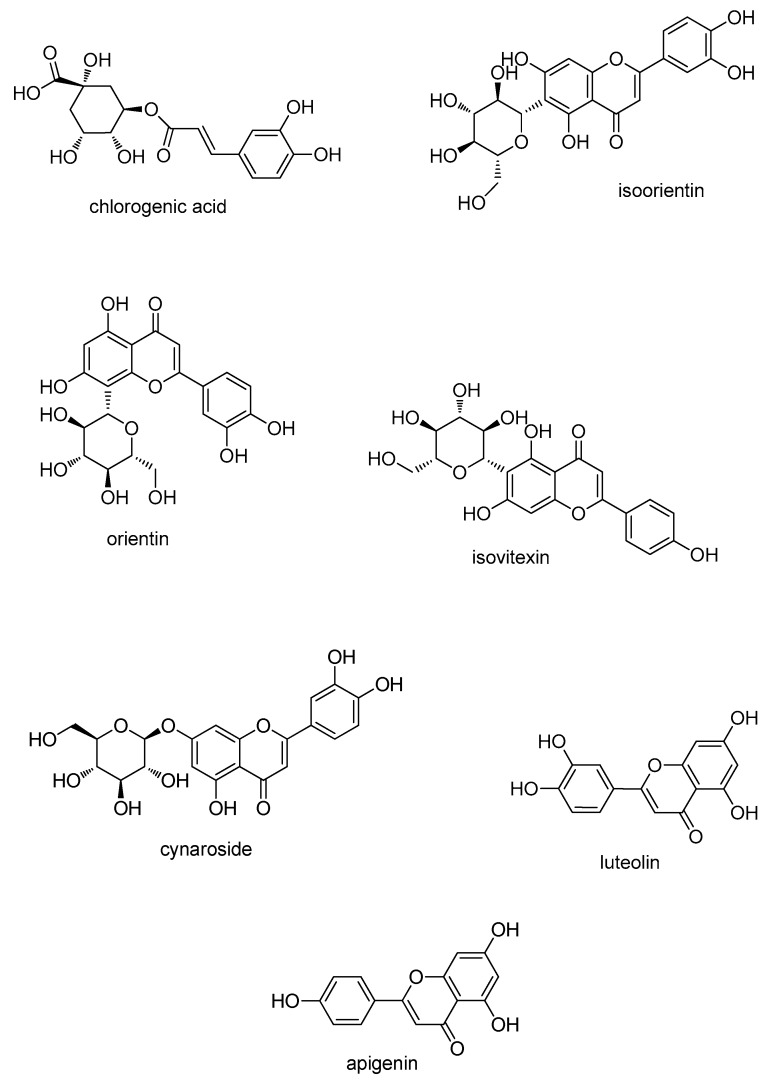
Chemical structures of the seven characteristic compounds.

Chlorogenic acid, isoorientin, and apigenin were purchased from Shanghai Natural Biological Technology Co., Ltd. (Shanghai, China). Iso-vitexin, cynaroside, and luteolin were obtained from Shanghai Yuanye Biological Technology Co., Ltd. (Shanghai, China). Orientin was bought from Chengdu Push Biological Technology Co., Ltd. (Chengdu, China). The purity of all standards was more than 98%, and their chemical structures are listed in Figure 5. DPPH was gained from Sigma-Aldrich (St. Louis, MO, USA). Trolox was from Beyotime Institute of Biotechnology Co., Ltd. (Shanghai, China). Ascorbic acid (VC) was ordered from Sinopharm Chemical Reagent Co., Ltd. (Shanghai, China). Diamonsil Plus C18 column (4.6 × 250 mm, 5 µm) was used. All liquid chromatography solvents (methanol, acetonitrile) were of HPLC grade, obtained from Anhui Fulltime Specialized Solvent and Reagent Co., Ltd. (Anqing, China), and used after filtration by a 0.45 µm organic membrane. All other solvents were of analytical grade and used without further purification.

### 4.2. Plant Materials

Twenty-six commercial herbal samples (Table 7) of *Phyllostachys nigra*, *Lophatherum gracile*, and *Pleioblastus amarus* were collected via purchase from commercial Chinese markets and authenticated by Dr. Long Song from Shanghai University of Traditional Chinese Medicine. The air-dried samples were smashed into powder (65 mesh) and stored in a desiccator.

### 4.3. Instruments and Chromatographic Conditions

HPLC analyses were primarily performed using an Agilent Technologies 1260 infinity pump and a 1260 infinity II ultraviolet (UV) detector (Palo Alto, CA, USA). The chromatography system consisted of a 1260 infinity quaternion liquid. A microplate spectrophotometer (Epoch 2, Biotek Instruments, Winooski, VT, USA) was used in the assay of the scavenging activity on the DPPH radical.

The separation was performed on a Diamonsil Plus C18 column (4.6 × 250 mm, 5 µm). The mobile phase was composed of 0.1% aqueous formic acid (A, pH 2.8) and acetonitrile (B), using a gradient elution of 10% B at 0–2 min, 10–30% B at 2–30 min, 30–70% B at 30–45 min, and 70–10% B at 55–60 min. The flow rate was set at 1.0 mL·min^−1^, with the temperature maintained at 30 °C. The injection volume was 10 µL and the detection wavelength was set at 350 nm.

### 4.4. Preparation of Solutions for HPLC Analysis

#### 4.4.1. Standard Solutions and Calibration Curves

The stock solution of each standard was prepared by dissolving single analyte in pure methanol at an appropriate concentration (CA, CYN, LUT, and API are about 0.5 mg·mL^−1^, and iso-ORI, ORI, and iso-VIT are about 1 mg·mL^−1^), and the preparations of ORI, iso-VIT, and API need to be pre-dissolved with dimethyl sulfoxide (DMSO). They may be precipitated when stored in a refrigerator, which can be solved by warming and ultrasound.

Standard solutions of CA, iso-ORI, ORI, iso-VIT, CYN, LUT, and API, in appropriate concentration ranges, were prepared by mixing each stock solution, and diluted by 50% methanol to obtain appropriate concentrations for calibration curves. The analyte peak area values were plotted against the corresponding concentrations of the analyte (expressed as µg·mL^−1^).

#### 4.4.2. Sample Solutions

Each powdered material (1.0 g) was accurately weighed and extracted with 25 mL of methanol under ultrasonication for 30 min followed by centrifugation. The supernatant solutions were filtered through a 0.22 µm filter membrane prior to injection.

### 4.5. Preparation of Solutions for DPPH Assay

Twenty-six extracts were prepared as the sample preparation for HPLC analysis. The seven characteristic compounds, as well as positive antioxidants including VC and trolox, were configured to be 20 mM with DMSO. All samples were evaluated by DPPH radical scavenging activity, and 50% methanol was used as the blank solution. For the DPPH assay, we have some modifications on the basis of reported methods [15]. Samples of 10 µL at various concentrations were added into 100 µL of DPPH solution (0.65 mM, methanol) and 140 µL of 50% methanol. After one hour of incubation at room temperature in the dark, the absorbance of mixtures (test samples, control samples, blank samples) was recorded at 517 nm. The scavenging rates (*SR*) were calculated based on the following formula:

*SR* (%) = 
100 × [1 − (*Abs _blank_* − *Abs _sample or control_*)/*Abs _blank_*], (2)

where *Abs _blank_* and *Abs _sample or control_* represent the absorbance of the blank sample, test sample, and control sample, respectively.

### 4.6. Statistical Analysis of Content and Antioxidant Activity

The correlation between the content of components and antioxidant activity of extracts was explored by reported multivariate regression analysis [33]. All statistical analyses were performed with the SPSS Statistics (SPSS vision 21, IBM, Camp Takajo, NY, USA).

## Figures and Tables

**Table 1:** The results of linearity, LOD, LOQ, and repeatability. RSD, relative standard deviation. CA, chlorogenic acid; iso-ORI, isoorientin; VIT, vitexin; CYN, Cynaroside; LUT, Luteolin; API, Apigenin.

Com.	Linear Range (µg·mL^−1^)	Linear Regression Equation (Y = a X + b) ^1^	*r*	LOD (µg·mL^−1^)	LOQ (µg·mL^−1^)	Repeatability (RSD, %, n = 5)
CA	1.15–73.80	Y = 13.39 X + 8.37	0.9999	0.14	0.42	3.04
iso-ORI	1.85‒118.40	Y = 29.14 X + 23.96	0.9999	0.08	0.17	2.86
ORI	0.85‒54.20	Y = 17.18 X + 5.86	0.9999	0.08	0.12	2.91
iso-VIT	1.74–111.40	Y = 26.87 X + 22.16	0.9999	0.06	0.11	2.71
CYN	0.32–20.24	Y = 28.32 X + 2.35	1.0000	0.09	0.18	2.09
LUT	1.54–24.72	Y = 39.79 X − 43.71	0.9998	0.19	0.58	0.14
API	1.28–10.22	Y = 47.77 X − 16.63	0.9996	0.35	1.04	0.19

^1^ Y refers to peak area and X refers to the concentration of compound.

**Table 2:** The results of the precision, stability, and recovery test.

Com.	Concentration (µg·mL^−1^)	Intraday Precision (RSD, %)	Stability (RSD, %)	Recovery Test				
				Initial (µg)	Added (µg)	Detected (µg)	Recovery (%)	RSD (%)
CA	4.612	0.14	0.73	5.45	2.72	8.37	107.02	0.86
	18.45	0.28			5.45	11.22	105.79	2.30
	73.80	1.13			8.18	14.19	106.88	0.53
iso-ORI	7.400	0.21	1.72	41.10	20.54	60.63	95.03	5.17
	29.60	0.21			41.09	79.28	92.90	2.21
	118.4	0.26			61.64	98.58	93.26	0.87
ORI	3.388	0.14	1.01	14.16	7.08	21.54	104.16	1.16
	13.55	0.19			14.16	29.12	105.64	0.84
	54.20	0.28			21.24	36.86	106.86	0.25
iso-VIT	6.962	0.10	2.18	20.77	10.38	31.12	99.71	2.59
	26.85	0.21			20.75	41.92	101.92	1.01
	111.4	0.28			31.11	53.55	105.30	0.05
CYN	1.265	0.22	2.01	1.26	0.63	1.92	103.30	2.61
	5.060	0.20			1.26	2.52	99.24	1.10
	20.24	0.39			1.89	3.15	99.59	0.92
LUT	1.545	1.07	2.58	0.45	0.22	0.69	107.40	1.56
	6.180	0.41			0.44	0.92	107.39	0.54
	24.72	0.28			0.67	1.18	109.26	0.41
API	0.6388	0.87	3.35	0.22	0.11	0.36	122.32	0.10
	2.555	0.15			0.22	0.48	112.14	0.39
	10.22	0.22			0.34	0.59	109.74	0.66

**Table 3:** The content results of the seven characteristic compounds.

No.	Botanical Name	Source	CA (µg·g^−1^)	iso-ORI (µg·g^−1^)	ORI (µg·g^−1^)	iso-VIT (µg·g^−1^)	CYN (µg·g^−1^)	LUT (µg·g^−1^)	API (µg·g^−1^)
1	*L. gracile*	Anhui	16.9 ± 0.9	1.4 ± 0	Nd	2.2 ± 0	Nd	13 ± 0.1	10.8 ± 0
2	*L. gracile*	Sichuan	7.5 ± 0.2	7.1 ± 0.1	326 ± 3.8	3.9 ± 0	92.6 ± 0.4	3.3 ± 0	11.4 ± 0.2
3	*L. gracile*	Zhejiang	8.1 ± 0.3	3.7 ± 0.1	214.1 ± 5	0.3 ± 0	21.8 ± 1.5	0.6 ± 0	6.5 ± 0.1
4	*L. gracile*	Guangdong	61.5 ± 0.6	7.6 ± 0.2	242.2 ± 6.8	4.6 ± 0.2	20.3 ± 1.2	0.4 ± 0	7.1 ± 0.1
5	*L. gracile*	Zhejiang	26.3 ± 1	22.4 ± 0.7	2.6 ± 0	5.6 ± 0.1	17.7 ± 0.8	54.1 ± 0.3	Nd
6	*L. gracile*	Zhejiang	175.5 ± 3.4	83.3 ± 7.3	6 ± 0.2	4.2 ± 0.1	48.1 ± 2.9	60.5 ± 0.3	23.1 ± 0
7	*L. gracile*	Hebei	35.6 ± 0.7	103.1 ± 10.1	1.5 ± 0.1	51.3 ± 3.7	38.5 ± 2.6	87.7 ± 1.6	23.6 ± 0
8	*P. amarus*	Hebei	175.9 ± 16.4	1923 ± 32.1	579.6 ± 10.6	897.1 ± 14.8	62.6 ± 1.1	21.6 ± 0.2	13.3 ± 0.1
9	*P. amarus*	Zhejiang	Nd	61.9 ± 2	17.4 ± 0.4	Nd	Nd	17.8 ± 0.2	6.6 ± 0.6
10	*P. amarus*	Guangdong	195.1 ± 2.9	440.3 ± 17.3	168.4 ± 3.4	98.9 ± 3.4	13.2 ± 0.4	16.7 ± 0.2	7.5 ± 0.3
11	*P. amarus*	Sichuan	Nd	52.5 ± 1.1	326.5 ± 2.8	0.4 ± 0	17 ± 0.4	49 ± 0	Nd
12	*P. amarus*	Sichuan	Nd	340.7 ± 8	107.3 ± 0.6	26.7 ± 0.6	101.7 ± 2.4	49.5 ± 0.1	30 ± 0.1
13	*P. amarus*	Jiangsu	12.2 ± 1.5	310.6 ± 12	Nd	46.3 ± 1.2	8.6 ± 0.5	18.6 ± 0.2	Nd
14	*P. amarus*	Sichuan	Nd	78.8 ± 3.1	473.7 ± 57.1	16 ± 0.5	Nd	Nd	Nd
15	*P. amarus*	Sichuan	30.4 ± 0.1	102.8 ± 1.7	571.4 ± 12.1	1.4 ± 0.1	18.8 ± 0.3	53.8 ± 0.2	Nd
16	*P. amarus*	Jiangxi	Nd	307.8 ± 8.6	136.8 ± 4.9	838 ± 17.6	21.2 ± 0.2	13.5 ± 0.1	11.8 ± 0.1
17	*P. amarus*	Sichuan	Nd	35.2 ± 1.8	226.7 ± 7.5	2.5 ± 0	11.3 ± 0.3	49 ± 0	Nd
18	*P. nigra*	Anhui	339.6 ± 11.7	488.6 ± 2.5	180 ± 3	390.8 ± 3.3	0.5 ± 0	15.3 ± 0	18.4 ± 0.1
19	*P. nigra*	Jiangxi	442.1 ± 8.5	304 ± 7.2	119.1 ± 4.6	404.2 ± 5.8	5.1 ± 0.5	15.5 ± 0	14.1 ± 0.3
20	*P. nigra*	Anhui	449 ± 41.4	185.1 ± 6.1	49.5 ± 3.9	347.2 ± 19.9	15.3 ± 1.4	14.3 ± 0.6	12.9 ± 0.3
21	*P. nigra*	Zhejiang	195 ± 1.4	607.3 ± 6.4	288.3 ± 4.4	317.5 ± 2.3	9.4 ± 0.4	49.2 ± 0.1	26.4 ± 0
22	*P. nigra*	Zhejiang	116.7 ± 5.1	863.3 ± 37.6	224.1 ± 9.7	386.1 ± 8.1	12.1 ± 0.5	50.8 ± 0.1	32 ± 0.4
23	*P. nigra*	Zhejiang	122.6 ± 3.8	1065.4 ± 50.1	377.5 ± 7.9	480.8 ± 14.7	11.7 ± 0.1	49.8 ± 0.1	33.6 ± 0.9
24	*P. nigra*	Sichuan	32 ± 0.2	269.2 ± 26.2	15.2 ± 1.4	33.2 ± 2.9	5.7 ± 0.1	52 ± 0.2	25.5 ± 0.1
25	*P. nigra*	Zhejiang	255.7 ± 2.4	414.3 ± 13.1	269.3 ± 8.4	301.6 ± 10.6	65.6 ± 1	51.6 ± 0.1	28 ± 0.2
26	*P. nigra*	Zhejiang	134.7 ± 3.2	12.5 ± 0.2	202.3 ± 9.3	12.4 ± 0.2	2.5 ± 0.2	0.4 ± 0	7.6 ± 0.1

**Table 4:** The dry weight and antioxidant inhibitory concentration (IC)_50_ values of 26 leaf extracts.

Sample No.	Botanical Name	Source	Dry Ointment Yield of Extracts (%)	IC_50_ Value (mg·mL^−1^)
1	*L. gracile*	Anhui	5.15 ± 0.11	4.09 ± 0.84
2	*L. gracile*	Sichuan	6.80 ± 0.09	6.69 ± 0.21
3	*L. gracile*	Zhejiang	5.51 ± 0.07	4.51 ± 0.03
4	*L. gracile*	Guangdong	5.74 ± 0.03	2.10 ± 0.06
5	*L. gracile*	Zhejiang	6.13 ± 0.07	10.17 ± 0.06
6	*L. gracile*	Zhejiang	8.06 ± 0.10	4.89 ± 0.58
7	*L. gracile*	Hebei	5.25 ± 0.04	3.06 ± 0.07
8	*P. amarus*	Hebei	8.93 ± 0.04	1.25 ± 0.12
9	*P. amarus*	Zhejiang	3.02 ± 0.05	2.11 ± 0.06
10	*P. amarus*	Guangdong	8.64 ± 0.04	2.18 ± 0.01
11	*P. amarus*	Sichuan	6.08 ± 0.03	3.09 ± 0.18
12	*P. amarus*	Sichuan	10.74 ± 1.17	3.25 ± 0.56
13	*P. amarus*	Jiangsu	6.67 ± 0.18	3.46 ± 0.39
14	*P. amarus*	Sichuan	8.04 ± 0.64	3.67 ± 0.25
15	*P. amarus*	Sichuan	8.36 ± 0.05	3.89 ± 0.07
16	*P. amarus*	Jiangxi	16.20 ± 0.84	4.48 ± 0.08
17	*P. amarus*	Sichuan	7.15 ± 0.07	5.07 ± 0.58
18	*P. nigra*	Anhui	15.10 ± 0.19	2.72 ± 0.06
19	*P. nigra*	Jiangxi	13.04 ± 0.05	2.02 ± 0.08
20	*P. nigra*	Anhui	12.42 ± 0.04	1.80 ± 0.02
21	*P. nigra*	Zhejiang	8.90 ± 0.45	1.59 ± 0.09
22	*P. nigra*	Zhejiang	8.90 ± 0.45	1.65 ± 0.08
23	*P. nigra*	Zhejiang	8.90 ± 0.45	1.85 ± 0.03
24	*P. nigra*	Sichuan	8.90 ± 0.45	2.63 ± 0.07
25	*P. nigra*	Zhejiang	9.43 ± 0.11	1.90 ± 0.14
26	*P. nigra*	Zhejiang	9.37 ± 0.16	1.65 ± 0.03

**Table 5:** The antioxidant IC_50_ values of compounds and the correlations between contents and IC_50_ values of extracts.

No.	Compound	IC_50_ Value (mM)	Correlation Coefficient *r*		
			PN	PA	LG
1	CA	1.04 ± 0.04	−0.023	−0.508 **	−0.169
2	Iso-ORI	0.81 ± 0.01	−0.171	−0.643 **	−0.181
3	ORI	0.84 ± 0.02	−0.501 **	−0.120	−0.133
4	Iso-VIT	14.5 ± 0.04	−0.193	−0.226	−0.293
5	CYN	0.43 ± 0.00	−0.164	−0.228	0.169
6	LUT	0.42 ± 0.01	0.021	0.431 *	0.139
7	API	Nd	−0.026	−0.264	−0.491 *
8	Trolox	0.97 ± 0.04	--	--	--
9	VC	0.93 ± 0.02	--	--	--

Nd, Not detected. --, None. * *p* < 0.05, ** *p* < 0.01.

**Table 6:** The antioxidant IC_50_ values of compounds and the correlations between contents and IC_50_ values of extracts.

Materials	Multiple Regression Equations ^2^	IC_50_ Value(mM)
*Lophatherum gracile*	Y = 8.413 − 21.269 X_ORI_ + 104.946 X_CYN_ − 383.105 X_API_	5.07 ± 0.26
*Phyllostachys nigra*	Y = 2.272 − 1.673 X_ORI_	3.25 ± 0.23
*Pleioblastus amarus*	Y = 3.279 − 3.855 X_iso-ORI_ + 1.468 X_Total_	1.98 ± 0.06

^2^ Y refers to IC_50_ values of an extract to scavenging DPPH free radical, and X means the content of a compound or the total content of seven characteristic compounds.

**Table 7:** Information sheet of twenty-six commercial herbal samples.

Botanical Name	Number	Source	Lot Number	Collection Date
*P. amarus*	1	Anguo Yikang Traditional Chinese Medicine Church, Hebei	XY170027	14 May 2017
	2	Anji, Zhejiang	XY170307	23 June 2017
	3	Meizhou, Guangdong	XY170308	23 June 2017
	4	Gaoxian, Sichuan	XY181247	26 March 2018
	5	Yibin, Sichuan	XY181235	16 March 2018
	6	Suqian, Jiangsu	XY181236	16 March 2018
	7	Shuanghe, Sichuan	XY181248	26 March 2018
	8	Meidong, Sichuan	XY181249	26 March 2018
	9	Yudu, Jiangxi	XY170502	14 December 2017
	10	Jiangan, Sichuan	XY181250	26 March 2018
*L. gracile*	1	Huoshan, Anhui	XY170499	10 December 2017
	2	Chengdu, Sichuan	XY170402	1 September 2017
	3	Shanghai kangqiao traditional Chinese medicine decoction pieces co. LTD	XY170487	21 September 2017
	4	Guangzhou, Guangdong	XY170489	5 November 2017
	5	Wenzhou, Zhejiang	XY181230	12 March 2018
	6	Lecheng, Zhejiang	XY181237	16 March 2018
	7	Anguo Oriental Medicine City, Hebei	XY170311	18 June 2017
*P. amarus*	1	Yueshan, Anhui	XY170524	23 December 2017
	2	Jiujiang, Jiangxi	XY170526	25 December 2017
	3	Anqing, Anhui	XY1700521	21 December 2017
	4	Changxing, Zhejiang	XY170492	6 November 2017
	5	Changxing, Zhejiang	XY181224	10 March 2018
	6	Zhejiang	XY181233	15 March 2018
	7	Qvzhou, Zhejiang (drying)	XY181242	18 March 2018
	8	Qvzhou, Zhejiang (air drying)	XY170027	18 March 2018
	9	Yibin, Sichuan	XY170307	16 March 2018
